# Relationship Between Dietary Omega-3 and Omega-6 Polyunsaturated Fatty Acids Level and Sarcopenia. A Meta-Analysis of Observational Studies

**DOI:** 10.3389/fnut.2021.738083

**Published:** 2022-01-12

**Authors:** Yi Zhang, Hongbin Guo, Jieyu Liang, Wenfeng Xiao, Yusheng Li

**Affiliations:** ^1^Department of Orthopaedics, Xiangya Hospital, Central South University, Changsha, China; ^2^National Clinical Research Center for Geriatric Disorders, Xiangya Hospital, Central South University, Changsha, China

**Keywords:** dietary omega-3 PUFAs, dietary omega-6 PUFAs, sarcopenia, meta-analysis, observational study

## Abstract

**Objective:** This study investigates the relationship between dietary omega-3 and omega-6 polyunsaturated fatty acids (PUFAs) levels and sarcopenia.

**Methods:** A comprehensive literature search in the databases of PubMed, Web of Science, and Embase (up to July 2021) were conducted to identify the observational studies on the relationship between dietary omega-3 and omega-6 PUFAs level and sarcopenia. The pooled odds ratio (OR) of sarcopenia for the highest vs. lowest dietary omega-3 and omega-6 PUFAs level and the standard mean difference (SMD) of dietary omega-3 and omega-6 PUFAs levels for sarcopenia vs. control subjects were calculated.

**Results:** A total of six studies were identified in this meta-analysis. The overall multi-variable adjusted OR showed that dietary omega-3 PUFAs level was inversely associated with sarcopenia (OR = 0.41, 95% CI: 0.26–0.65; *P* = 0.0001). Moreover, the overall combined SMD showed that the dietary omega-3 PUFAs level in sarcopenia was lower than that in control subjects (SMD = −0.19, 95% CI: −0.32 to −0.07; *P* = 0.002). With regard to dietary omega-6 PUFAs level, the overall multi-variable adjusted OR suggested no significant relationship between dietary omega-6 PUFAs level and sarcopenia (OR = 0.64, 95% CI: 0.33–1.24; *P* = 0.19). However, the overall combined SMD showed that the dietary omega-6 PUFAs level in sarcopenia was slightly lower than that in control subjects (SMD = −0.15, 95% CI: −0.27 to −0.02; *P* = 0.02).

**Conclusion:** Our results suggested that the dietary omega-3 PUFAs level was inversely associated with sarcopenia. However, current evidence is still insufficient to demonstrate the definite relationship between dietary omega-6 PUFAs levels and sarcopenia. More well-designed prospective cohort studies with the dietary omega-3/omega-6 PUFAs ratio are still needed.

## Introduction

Sarcopenia, an age-related degenerative disorder, is characterized by the reduction of muscle mass, strength, and physical performance (1). Sarcopenia is considered to be associated with frailty, mobility limitation, and mortality in elderly populations (2). With the increase in life expectancy, sarcopenia has become a serious health issue in the elderly. Generally speaking, the pathology of sarcopenia is related to aging, body composition, exercise, and inflammation status (3–5). In addition, the dietary factors have also entered the researchers' field of vision (6). Thus, the identification of modifiable dietary factors for sarcopenia appears to be important for its management.

Essential fatty acids are composed of two kinds of polyunsaturated fatty acids (PUFAs), omega-3 and omega-6 PUFAs. These two important nutritional bioactive compounds often have opposing physiological functions (7). Some epidemiological studies indicated that dietary omega-3 PUFAs level was inversely associated with cardiovascular disease (8), metabolic syndrome (9), and fractures (10). Importantly, PUFAs were also associated with the hypertrophy of muscle (11). A meta-analysis of randomized controlled trials has demonstrated that omega-3 PUFAs supplementation could increase muscle mass and strength in the elderly population (12). Moreover, omega-3 PUFAs could also modulate the molecules related to sarcopenia in experimental animal models (13–15). Taken together, the dietary omega-3 PUFAs level seems to be inversely associated with sarcopenia.

To our best knowledge, several observational studies have examined the relationship between dietary omega-3 and omega-6 PUFAs levels and sarcopenia. However, their results are still controversial (16–21). Therefore, this meta-analysis of observational studies was systematically performed to investigate the above issues further.

## Methods

### Search Strategy

This meta-analysis was performed according to the Preferred Reporting Items for Systematic review and Meta-analyses (PRISMA) guidelines (22). Using a series of logic combinations of sarcopenia (“sarcopenia,” “sarcopenic”) and fatty acids (“fatty acids,” “fatty acids”), the electronic databases of PubMed, Web of Science, and Embase were searched up to July 2021. No language restrictions were set in the search strategy. The titles and abstracts were first screened and then the full articles were read to identify eligible studies.

### Study Selection

The title and abstract screening of relevant articles was done separately by two researchers (YZ and YSL) to identify eligible studies for inclusion. The potentially eligible articles were required to meet the following criteria: (1) observational studies; (2) the exposure of interest was the dietary omega-3 or omega-6 PUFAs level; (3) the outcomes included sarcopenia; (4) Odds ratio (OR) with 95% CI or the standard mean difference (SMD) of dietary omega-3 and omega-6 PUFAs level for sarcopenia vs. control subjects were reported. The exclusion criteria were listed as follows: (1) duplicated or irrelevant articles; (2) reviews, letters, or case reports; (3) randomized controlled trials; and (4) non-human studies.

### Data Extraction

Data extraction was conducted by two independent reviewers (YZ and YSL); disagreements were resolved by consensus. The first author, year of publication, location, age, gender, sample size, adjustment, exposure assessment, category of exposure, effect estimates, and the diagnostic criteria of sarcopenia were extracted. The corresponding effect estimates adjusted for the maximum number of confounding variables with corresponding 95% CIs for the highest vs. lowest level were calculated. Moreover, the dietary omega-3 and omega-6 PUFAs levels (mean ± SD) for sarcopenia vs. control subjects were also extracted to calculate the SMD.

### Statistical Analysis

The OR for sarcopenia and SMD for dietary omega-3 and omega-6 PUFAs levels were the outcome measures in our study. The *I*^2^ statistic, which measures the percentage of the total variation across studies due to heterogeneity, was examined (*I*^2^ > 50% was considered heterogeneity). If significant heterogeneity was observed among studies, the random-effects model was used; otherwise, the fixed-effects model was acceptable. Begg's tests were performed to assess the publication bias (23), and statistical analyses were performed using STATA version 11 (StataCorp LP, College Station, TX, USA). A value of *p* ≤ 0.05 was accepted as statistically significant. A subgroup analysis for a geographical region, gender, sample size, exposure assessment, adjustment of BMI, energy intake, and physical activity was conducted for OR analysis. The subgroup analysis for dietary omega-6 PUFAs level was not employed since only 3 studies were available. In addition, a subgroup analysis for a geographical region, sample size, and exposure assessment was also performed for SMD analysis. Since the SD for omega-3 PUFAs consumption was reported as “0.0” in Yang's study (19), we excluded this study in SMD analysis. In addition, a sensitivity analysis was conducted to determine whether an individual study may affect the pooled result.

## Results

### Study Identification and Selection

**Figure 1:**
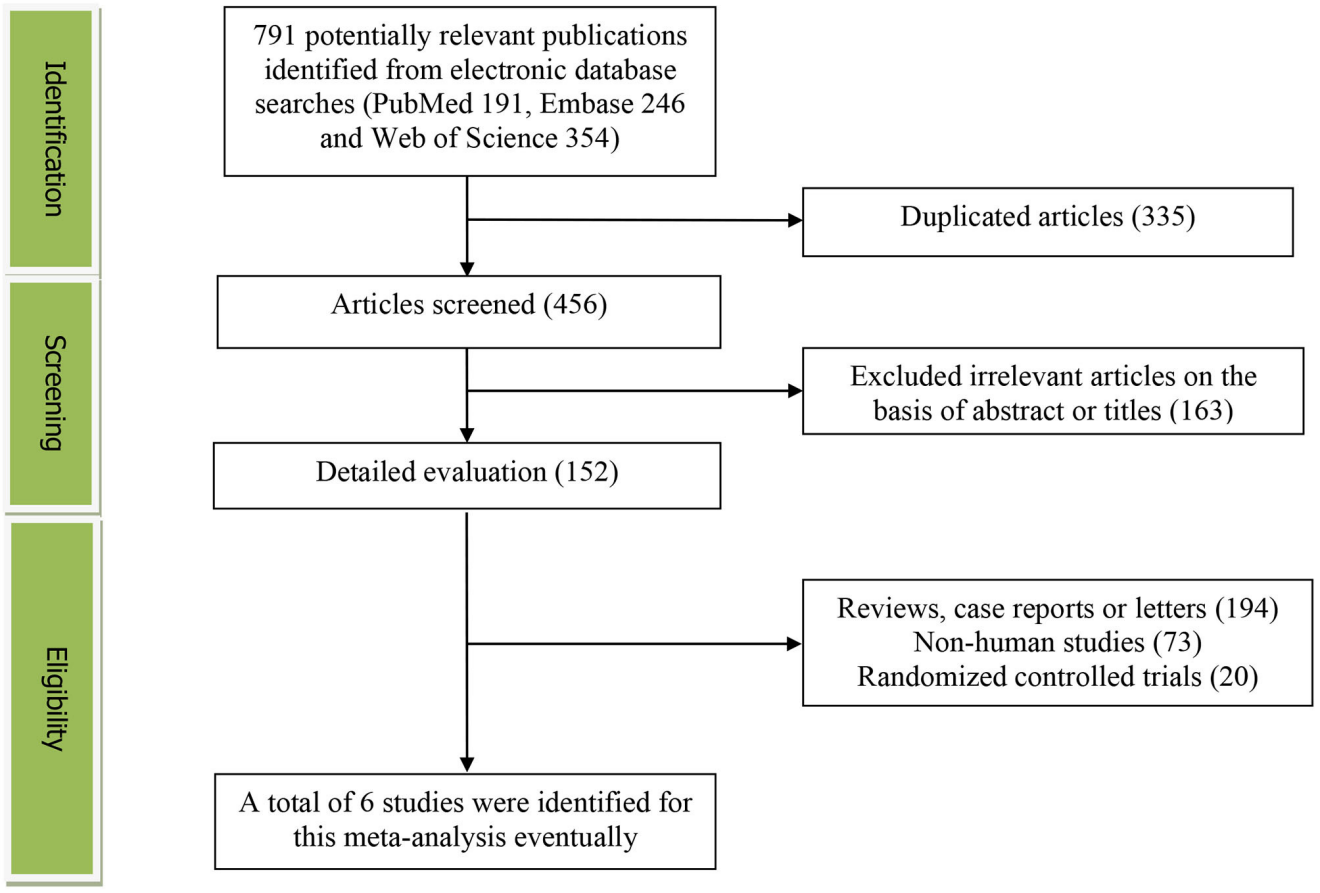
The detailed flow diagram of the study identification and selection in this meta-analysis.

The detailed flow diagram of the articles included in this meta-analysis was presented in Figure 1. A total of 791 potentially relevant articles (PubMed: 191, Embase: 246, and Web of Science: 354) were yielded during initial literature searches. After eliminating 335 duplicated articles, 456 articles were screened by titles and abstracts. One hundred and sixty-three irrelevant studies, 194 reviews, case reports or letters, 73 non-human studies, and 20 randomized controlled trials studies were removed. Eventually, a total of 6 articles were identified for this meta-analysis (17–22).

### Study Characteristics

The main characteristics of the included studies were presented in Table 1. These studies were published between 2016 and 2021, which included six studies. three studies were performed in Asia (Japan and Korea) (17, 19, 21), and the other 3 ones were conducted in the Netherlands (16), Australia (18), and Brazil (20). Five studies included both male and female participants (16, 17, 19–21), whereas 1 study included only male participants (18). The sample size ranged from 125 to 3,815. The dietary PUFAs level was assessed by food-frequency questionnaire (FFQ) in five studies (16–19, 21) and 24 h recall method in 1 study (20). The diagnostic criteria of sarcopenia are European Working Group on Sarcopenia (EWGSOP) (16, 20), Asian Working Group for Sarcopenia (AWGS) (19, 21), Foundation for the National Institutes of Health (FNIH) (18), and Japan Society of Hepatology guidelines for sarcopenia (JSHS) (17), respectively.

**Table 1:** Characteristics of the individual studies included in this meta-analysis.

**First author year of publication**	**Location**	**Age**	**Gender**	**Sample size**	**Adjustments**	**Exposure assessment**	**Category of exposure**	**Effect estimates (OR or SMD)**	**Diagnostic criteria of sarcopenia**
Borg (16)	Netherlands	≥65	Both	227	NA	104-item FFQ	Control subjects Sarcopenia subjects Control subjects Sarcopenia subjects	Omega-3 PUFAs 7.14 (6.96, 7.32) 6.98 (6.70, 7.26) Omega-6 PUFAs 28.0 (27.7, 28.3) 27.9 (27.5, 28.3)	EWGSOP
Das (18)	Australia	≥75	Male	794	Age, BMI, marital status, living arrangement, income, smoking status, MMSE score, alcohol intake, SRH, meal service, able to shop for groceries, meal preparation, no of co-morbidities, PASE and energy	111-item FFQ	Omega-3 PUFAs Quartiles 1 Quartiles 2 Quartiles 3 Quartiles 4 Omega-6 PUFAs Quartiles 1 Quartiles 2 Quartiles 3 Quartiles 4 Control subjects Sarcopenia subjects Control subjects Sarcopenia subjects	1.00 0.86 (0.40, 1.83) 0.70 (0.30, 1.63) 0.45 (0.21, 0.95) 1.00 0.58 (0.25, 1.33) 0.45 (0.19, 1.04) 0.36 (0.17, 0.78) Omega-3 PUFAs 1.30 (1.23, 1.37) 1.10 (0.96, 1.24) Omega-6 PUFAs 10.10 (9.67, 10.53) 8.70 (7.70, 9.70)	FNIH
Okamura (17)	Japan	≥65	Both	342	Age, sex, exercise, smoking status, diabetes duration, Hemoglobin A1c, energy intake, protein intake, fat intake	58-item FFQ	Omega-3 PUFAs Tertiles 1 Tertiles 2 Tertiles 3 Control subjects Sarcopenia subjects Control subjects Sarcopenia subjects	1.0 0.62 (0.20, 3.16) 0.10 (0.02, 0.64) Omega-3 PUFAs 3.00 (2.86, 3.14) 2.60 (2.30, 2.90) Omega-6 PUFAs 10.10 (9.64, 10.56) 8.80 (7.78, 9.82)	JSHS
Yang (19)	Korea	≥60	Both	3,815	Age, BMI, total cholesterol, systolic blood pressure, fasting plasma glucose, AST, hsCRP, current smoking status, heavy alcohol intake, economic status, marital status, education duration, occupation, sufficient physical activity, history of diabetes mellitus, history of hypertension and protein intake	FFQ	Male Omega-3 PUFAs Quartiles 1 Quartiles 2 Quartiles 3 Quartiles 4 Female Omega-3 PUFAs Quartiles 1 Quartiles 2 Quartiles 3 Quartiles 4 Male Control subjects Sarcopenia subjects Female Control subjects Sarcopenia subjects	1.00 1.56 (0.80, 3.05) 1.25 (0.61, 2.53) 0.92 (0.46, 1.86) 1.00 0.66 (0.38, 1.16) 0.64 (0.37, 1.10) 0.25 (0.11, 0.53) Omega-6 PUFAs 3.70 (3.50, 3.90) 3.90 (3.50, 4.30) Omega-6 PUFAs 4.00 (3.80, 4.20) 3.80 (3.41, 4.19)	AWGS
Reis (20)	Brazil	48	Both	125	Sex, age, weight, waist circumference, energy intake, glomerular filtration rate, physical activity, C-reactive protein, use of immunosuppressive and corticoids drugs: calcineurin inhibitor, cell proliferation and mTOR inhibitors, prednisone and caloric intake misreporting	24 h recall	Omega-3 PUFAs Non-consumer Consumer Omega-6 PUFAs Non-consumer Consumer Control subjects Sarcopenia subjects Control subjects Sarcopenia subjects	1.00 0.55 (0.45, 0.93) 1.00 0.97 (0.87, 1.07) Omega-3 PUFAs 1.40 (0.70, 2.10) 1.40 (0.50, 2.30) Omega-6 PUFAs 14.3 (12.8, 15.8) 12.6 (10.2, 15.0)	EWGSOP
Otsuka (21)	Japan	≥60	Both	1,345	Age, sex, BMI, residence area, current smoking habit, current alcohol drinking habit, and total caloric intake	56-item FFQ	Omega-3 PUFAs Quartiles 1 Quartiles 2 Quartiles 3 Quartiles 4 Omega-6 PUFAs Quartiles 1 Quartiles 2 Quartiles 3 Quartiles 4 Control subjects Sarcopenia subjects Control subjects Sarcopenia subjects	1.00 1.19 (0.54, 2.63) 0.96 (0.41, 2.23) 0.28 (0.09, 0.89) 1.00 1.65 (0.77, 3.51) (0.42, 2.39) 0.39 (0.12, 1.26) Omega-3 PUFAs 3.10 (3.02, 3.18) 2.90 (2.58, 3.22) Omega-6 PUFAs 10.3 (10.1, 10.5) 9.0 (8.3, 9.7)	AWGS

### OR of Sarcopenia for the Highest vs. Lowest Dietary Omega-3 PUFAs Category

**Figure 2:**
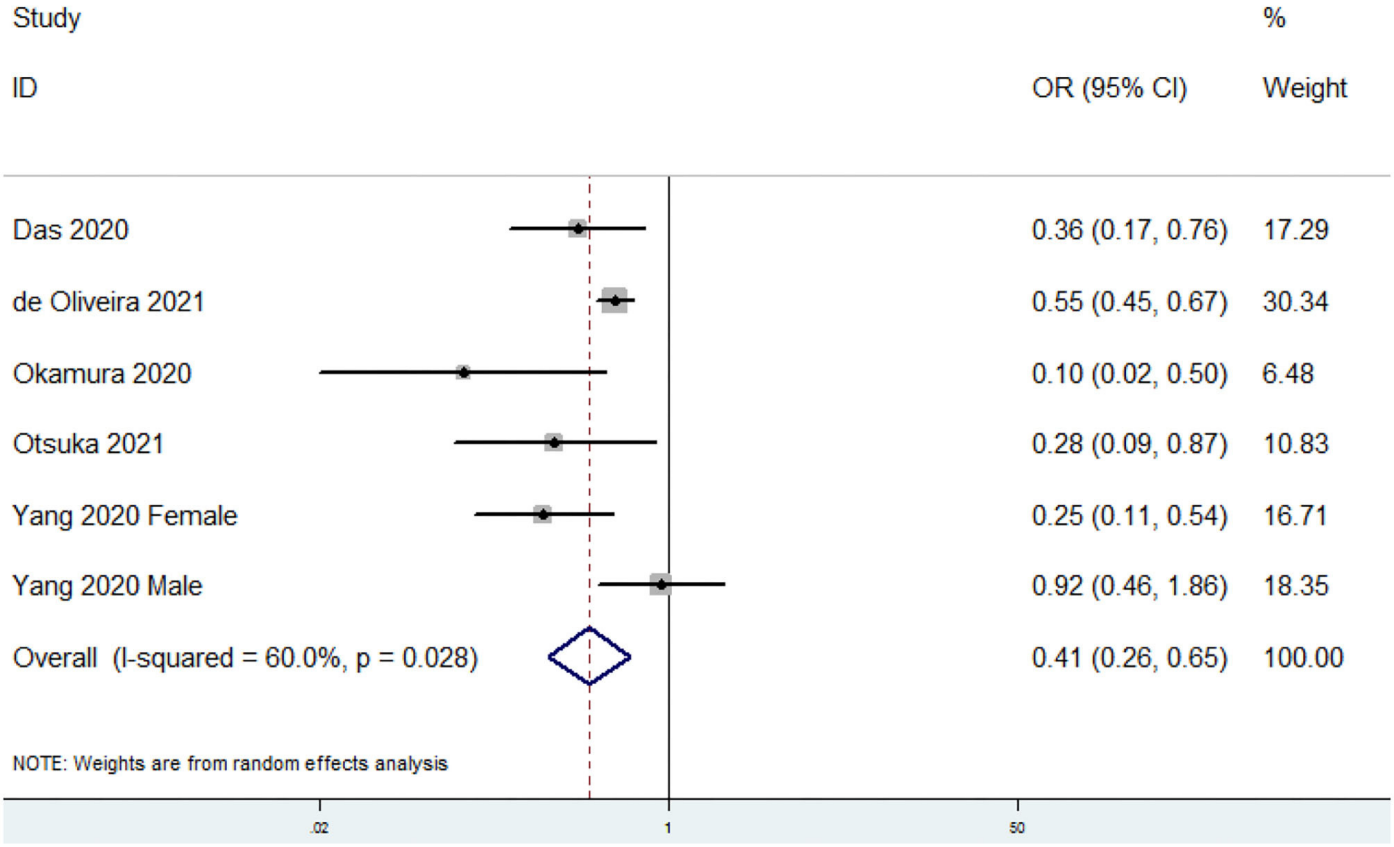
Forest plot of meta-analysis: Overall multi-variable adjusted OR of sarcopenia for the highest vs. lowest category of dietary omega-3 PUFAs level.

The overall multi-variable adjusted OR showed that the dietary omega-3 PUFAs level was inversely associated with sarcopenia (OR = 0.41, 95% CI: 0.26–0.65; *P* < 0.001) (Figure 2). A substantial level of heterogeneity was found among various studies (*P* = 0.028, *I*^2^ = 60%). Begg's rank-correlation test showed no evidence of publication bias (*P* = 0.06). The results of subgroup analysis were presented in Table 2. The above findings were confirmed in women (OR = 0.25, 95% CI: 0.11–0.54), >500 sample-sized (OR = 0.41, 95% CI: 0.21–0.77; *P* = 0.006), adjustment of BMI (OR = 0.89, 95% CI: 0.86–0.93; *P* < 0.001), and energy intake (OR = 0.38, 95% CI: 0.19–0.75; *P* = 0.005) studies, but not in men (OR = 0.58, 95% CI: 0.23–1.47; *P* = 0.25), <500 sample-sized (OR = 0.28, 95% CI: 0.06–1.45; *P* = 0.13), unadjustment of BMI (OR = 0.28, 95% CI: 0.06–1.45; *P* = 0.13), and energy intake (OR = 0.42, 95% CI: 0.17–1.05; *P* = 0.06) studies.

**Table 2:** Subgroup analysis of sarcopenia for the highest vs. lowest dietary omega-3 polyunsaturated fatty acids (PUFAs) category.

**Stratification**	**Number of studies**	**Pooled OR**	**95% CI**	***P*** **-value**	**Heterogeneity**
All studies	5	0.41	0.26, 0.65	*P* <0.001	*P* = 0.03; *I*^2^ = 60%
Geographical region					
Asia	3	0.33	0.13, 0.80	*P* = 0.01	*P* = 0.02; *I*^2^ = 71%
Non-Asia	2	0.53	0.44, 0.65	*P* <0.001	*P* = 0.28; *I*^2^ = 13%
Gender					
Male	2	0.58	0.23, 1.47	*P* = 0.25	*P* = 0.07; *I*^2^ = 69%
Female	1	0.25	0.11, 0.54	/	/
Sample size					
<500	2	0.28	0.06, 1.45	*P* = 0.13	*P* = 0.04; *I*^2^ = 76%
>500	3	0.41	0.21, 0.77	*P* = 0.006	*P* = 0.06; *I*^2^ = 59%
Exposure assessment					
FFQ	4	0.35	0.18, 0.67	*P* = 0.002	*P* = 0.04; *I*^2^ = 61%
24 h recall	1	0.55	0.45, 0.67	/	/
Diagnostic criteria of sarcopenia					
AWGS	2	0.42	0.17, 1.05	*P* = 0.06	*P* = 0.03; *I*^2^ = 71%
Other	3	0.38	0.19, 0.75	*P* = 0.005	*P* = 0.07; *I*^2^ = 62%
Adjustment of BMI					
Adjusted	3	0.41	0.21, 0.77	*P* = 0.006	*P* = 0.06; *I*^2^ = 59%
Unadjusted	2	0.28	0.06, 1.45	*P* =0.13	*P* = 0.04; *I*^2^ = 76%
Adjustment of physical activity					
Adjusted	2	0.52	0.30, 0.91	*P* = 0.02	*P* = 0.05; *I*^2^ = 68%
Unadjusted	3	0.28	0.16, 0.51	*P* <0.001	*P* = 0.37; *I*^2^ = 0%
Adjustment of energy intake					
Adjusted	3	0.38	0.19, 0.75	*P* = 0.005	*P* = 0.07; *I*^2^ = 62%
Unadjusted	2	0.42	0.17, 1.05	*P* = 0.06	*P* = 0.03; *I*^2^ = 71%
Adjustment of co-morbidities					
Adjusted	3	0.35	0.16, 0.78	*P* = 0.01	*P* = 0.02; *I*^2^ = 70%
Unadjusted	2	0.54	0.44, 0.66	*P* <0.001	*P* = 0.25; *I*^2^ = 24%

### SMD of the Dietary Omega-3 PUFAs Level for Sarcopenia vs. Control Subjects

**Figure 3:**
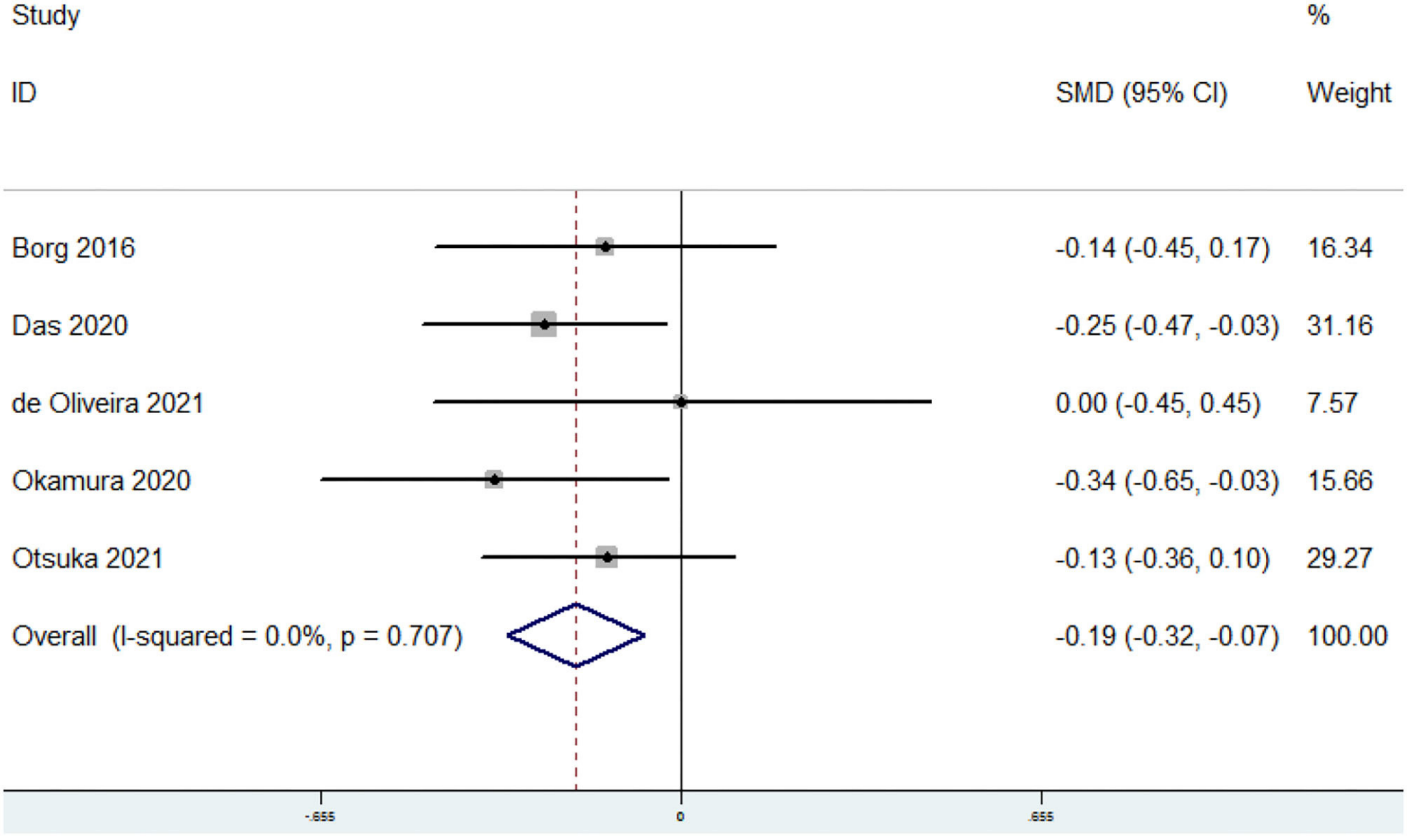
Forest plot of meta-analysis: SMD of dietary omega-3 PUFAs level for sarcopenia vs. control subjects.

The overall combined SMD showed that the dietary omega-3 PUFAs level in sarcopenia was lower than that in control subjects (SMD = −0.19, 95% CI: −0.32 to −0.07; *P* = 0.002) (Figure 3). No substantial level of heterogeneity was found among various studies (*P* = 0.707, *I*^2^ = 0%). Begg's rank-correlation test showed no evidence of publication bias (*P* = 0.806). The results of subgroup analysis were showed in Table 3. The above findings were confirmed in FFQ (SMD = −0.21, 95% CI: −0.34 to −0.08; *P* = 0.002), but not 24 h recall method (SMD = 0, 95% CI: −0.45 to 0.45) studies.

**Table 3:** Subgroup analysis for standard mean difference (SMD) of dietary omega-3 PUFAs level in sarcopenia vs. control subject.

**Stratification**	**Number of studies**	**Pooled OR**	**95% CI**	***P*** **-value**	**Heterogeneity**
All studies	5	−0.19	−0.32, −0.07	*P* = 0.002	*P* = 0.71; *I*^2^ = 0%
Geographical region					
Asia	2	−0.21	−0.39, −0.02	*P* = 0.03	*P* = 0.30; *I*^2^ = 6%
Non-Asia	3	−0.18	−0.35, −0.01	*P* = 0.03	*P* = 0.59; *I*^2^ = 0%
Sample size					
<500	3	−0.19	−0.39, 0.01	*P* = 0.06	*P* = 0.44; *I*^2^ = 0%
>500	2	−0.19	−0.35, −0.03	*P* = 0.02	*P* = 0.48; *I*^2^ = 0%
Exposure assessment					
FFQ	4	−0.21	−0.34, −0.08	*P* = 0.002	*P* = 0.71; *I*^2^ = 0%
24 h recall	1	0.00	−0.45, 0.45	/	/

### OR of Sarcopenia for the Highest vs. Lowest Dietary Omega-6 PUFAs Category

**Figure 4:**
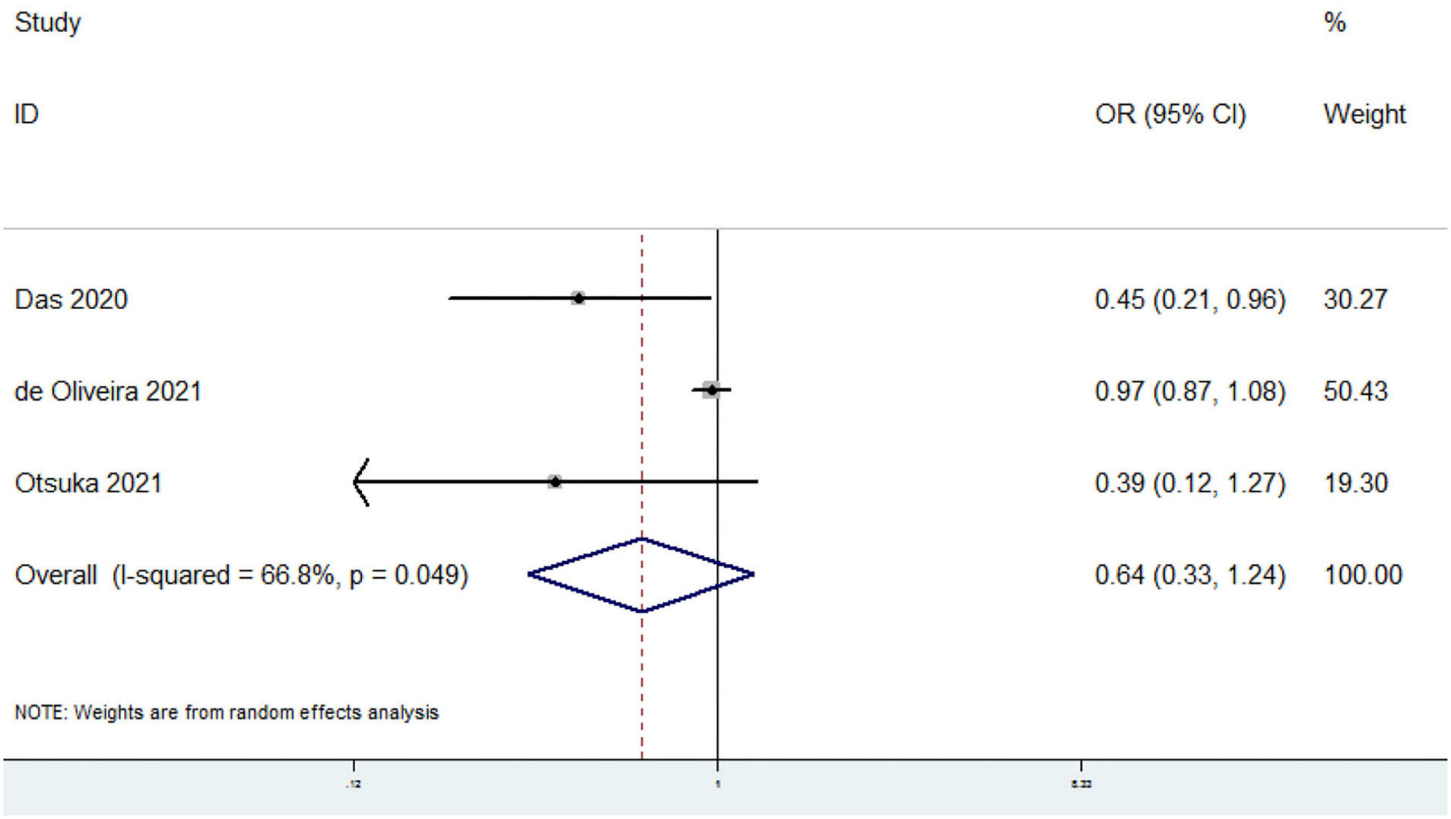
Forest plot of meta-analysis: Overall multi-variable adjusted OR of sarcopenia for the highest vs. lowest category of dietary omega-6 PUFAs level.

The overall multi-variable adjusted OR suggested no significant relationship between dietary omega-6 PUFAs level and sarcopenia (OR = 0.64, 95% CI: 0.33 to 1.24; *P* = 0.19) (Figure 4). A substantial level of heterogeneity was found among various studies (*P* = 0.049, *I*^2^ = 66.8%). Begg's rank-correlation test showed no evidence of publication bias (*P* = 1).

### SMD of the Dietary Omega-6 PUFAs Level for Sarcopenia vs. Control Subjects

**Figure 5:**
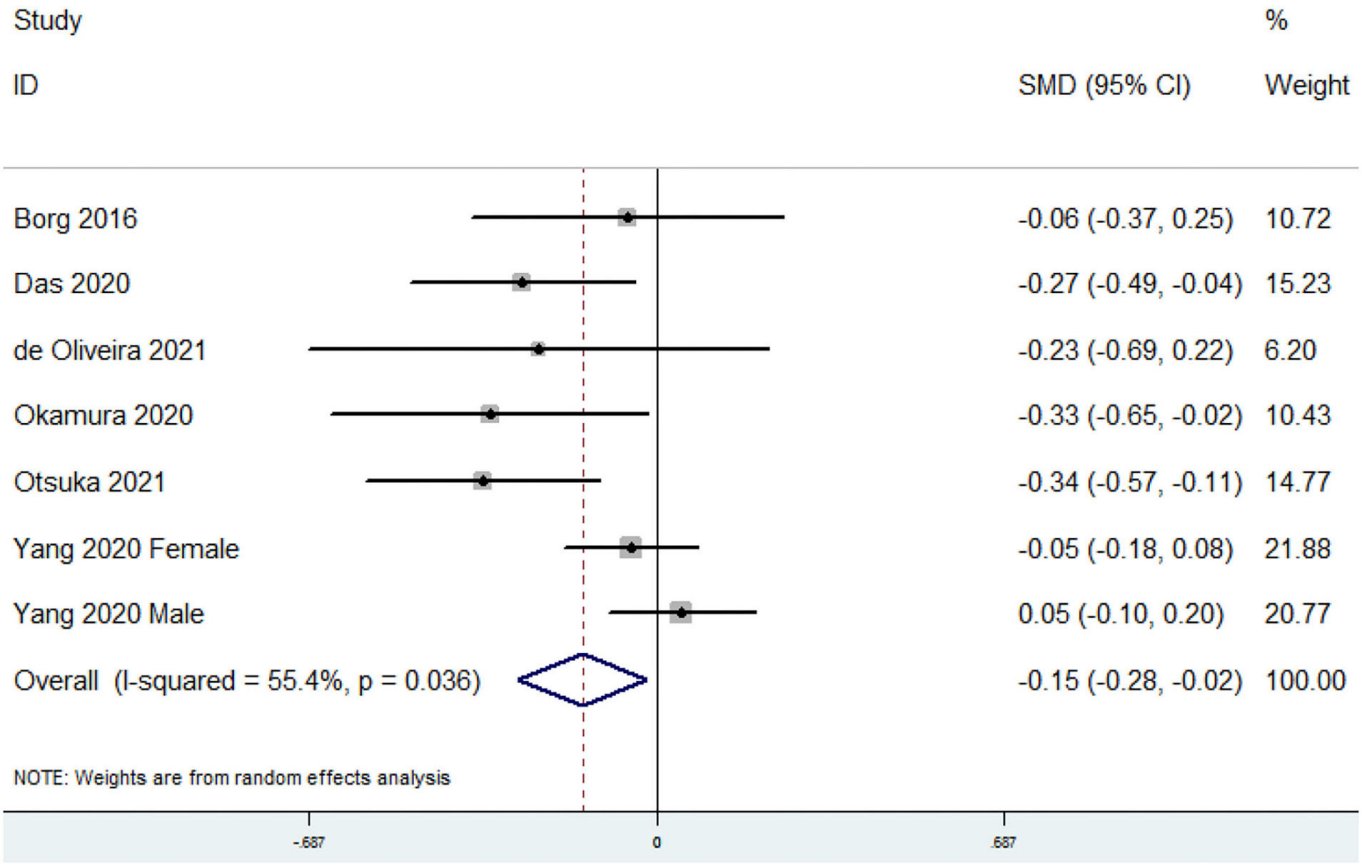
Forest plot of meta-analysis: SMD of dietary omega-6 PUFAs level for sarcopenia vs. control subjects.

The overall combined SMD showed that the dietary omega-6 PUFAs level in sarcopenia was lower than that in control subjects (SMD = −0.15, 95% CI: −0.28 to −0.02; *P* = 0.023) (Figure 5). A substantial level of heterogeneity was found among various studies (*P* = 0.036, *I*^2^ = 55.4%). Begg's rank-correlation test showed no evidence of publication bias (*P* = 0.548). The results of subgroup analysis were showed in Table 4. The above findings were confirmed in FFQ (SMD = −0.14, 95% CI: −0.28 to −0.01; *P* = 0.04), but not 24 h recall method (SMD = −0.23, 95% CI: −0.69 to 0.22) studies.

**Table 4:** Subgroup analysis for SMD of dietary omega-6 PUFAs level in sarcopenia vs. control subject.

**Stratification**	**Number of studies**	**Pooled OR**	**95% CI**	***P*** **-value**	**Heterogeneity**
All studies	6	−0.15	−0.27, −0.02	*P* = 0.02	*P* = 0.04; *I*^2^ = 55%
Geographical region					
Asia	3	−0.14	−0.31, 0.04	*P* = 0.13	*P* = 0.01; *I*^2^ = 72%
Non-Asia	3	−0.20	−0.37, −0.03	*P* = 0.02	*P* = 0.56; *I*^2^ = 0%
Sample size					
<500	3	−0.20	−0.40, 0.00	*P* = 0.05	*P* = 0.48; *I*^2^ = 0%
>500	3	−0.13	−0.30, 0.04	*P* = 0.12	*P* = 0.01; *I*^2^ = 72%
Exposure assessment					
FFQ	5	−0.14	−0.28, −0.01	*P* = 0.04	*P* = 0.02; *I*^2^ = 62%
24 h recall	1	−0.23	−0.69, 0.22	/	/

### Sensitivity Analysis

The sensitivity analysis showed only minimal changes in the pooled estimate when any study was excluded from the meta-analysis. It showed that no individual study had an excessive influence on these robust aggregate results (data not shown).

## Discussions

In the present meta-analysis, a total of 6 studies were identified for examination, and the pooled analysis showed that the dietary omega-3 PUFAs level was inversely associated with sarcopenia.

The potential negative association between dietary omega-3 PUFAs level and sarcopenia may be explained as follow. First, omega-3 PUFAs could activate the mammalian target of rapamycin/ribosomal protein kinase S6 (mTORp/70s6k) signaling pathway and then improve the anabolism of net muscle protein. The sensitivity of responses to anabolic stimuli (enhanced protein intake, resistance exercise, and insulin) could therefore be increased (24, 25). Second, omega-3 PUFAs could inhibit the synthesis of Prostaglandin I2 (PGI2) at the level of cyclooxygenases (COX) and subsequently inhibit lipogenesis (26–28). Third, omega-3 PUFAs could attenuate oxidative stress and suppress muscle atrophy by suppressing the forkhead box-containing protein O1 (foxo1) pathway (29, 30). Fourth, animal studies have demonstrated that omega-3 PUFAs alone or combined with cholecalciferol or menaquinone-7 intervention could significantly modulate the molecules related to sarcopenia (13–15).

Recently, our topic has been discussed by a meta-analysis of randomized controlled trials, which showed a beneficial effect of omega-3 PUFAs supplementation on muscle mass and walking speed in the elderly population (especially for those consuming more than 2 g/day) (12). Indeed, our results showed an inverse relationship between dietary omega-3 PUFAs level and sarcopenia. Moreover, another recent meta-analysis has compared the nutrient intake among the elderly with and without sarcopenia (31). It showed that the saturated fatty acids intake in sarcopenia subjects was lower than that in control. However, PUFAs were not considered. Taken together, our study was a perfect addition to current evidence.

So far, the circulating omega-3 PUFAs level in sarcopenia has also been investigated in several studies. Borg et al. indicated that a lower circulating level of eicosapentaenoic acid (EPA) was associated with sarcopenia (32). Jang et al. showed that the serum omega-3 PUFAs level was positively associated with muscle strength (33). Moreover, Murphy showed that the depleted plasma omega-3 PUFAs in cancer patients with sarcopenia may contribute to accelerate the rates of muscle loss (34). In addition, Belury et al. also found that the lean mass and grip strength in women with breast cancer was positively associated with the level of erythrocyte omega-3 PUFAs (35). On the other hand, the bioavailability of omega-3 PUFAs is commonly ignored in defeating disorders. The biological effect of omega-3 PUFAs is determined by the formation of phospholipids and the regulation of membrane properties. The omega-3 PUFAs content in erythrocyte membranes (36) and fatty acids-based membrane lipidomics (37) are considered as reliable indicators for omega-3 PUFAs intake. However, FFQ could not reflect the biological effective quantity of omega-3 PUFAs. Above all, further studies on the bioavailability of omega-3 PUFAs are still needed.

Interestingly, the inverse relationship between dietary omega-3 PUFAs levels and sarcopenia was only obtained in women, but not in men. Although the number of studies with gender specification is rather limited, some genetic gender differences may be associated with the diet-related pathology of sarcopenia. In addition, the inconsistent result with regard to the adjustment of BMI and energy intake was also acquired. Indeed, BMI and energy intake were reported to be associated with omega-3 PUFAs and sarcopenia (38–41), which indicated that they should be considered as confounding factors in further study. Moreover, the significant findings were disappeared in <500 sample-sized studies, suggesting that a large sample-sized study may be more reliable to address the issues. Regarding the diagnostic criteria of sarcopenia, the corresponding result was lost in AWGS but existed in others. However, the distribution of diagnostic criteria is scattered (4 criteria in 6 studies), which may influence the reliability of this subgroup analysis. Above all, further large well-designed studies are still needed to address the issues above.

The metabolites of omega-6 fatty acids are pro-inflammatory/pro-aggregatory agents (42), which may contribute to the risk of all-cause or coronary heart disorder mortality and cardiovascular events (43). Indeed, omega-3 and omega-6 fatty acids are supposed to balance each other when they are consumed in the diet at a ratio of around 1 to 1 (44). The decrease in omega-3/omega-6 PUFAs ratio could shift the balance into a pro-inflammatory/pro-aggregatory state (44). Thus, the omega-3/omega-6 PUFAs ratio may be more useful and reliable than omega-6 PUFAs alone, which may also partly account for the inconsistent results with regard to its OR and SMD analysis. Taken together, more studies with the dietary omega-3/omega-6 PUFAs ratio are needed to address the issues above.

Our study has several strengths. First, this is the first meta-analysis of observational studies on the relationship between dietary omega-3 and omega-6 PUFAs levels and sarcopenia based on the most comprehensive literature search to date. Second, all included studies were published in recent years, suggesting a potential novel topic in our study. Third, our results are consistent with the corresponding clinical and experimental research, which may be helpful to better consider the effect of diet on sarcopenia.

The limitations of this meta-analysis should also be notified. First, the substantial level of heterogeneity might have distorted the results. Second, due to the limitation of relevant literature, only 6 studies were identified totally. Third, the classification of exposure may also vary greatly among individuals. Fourth, the diagnostic criteria of sarcopenia and selection of adjusted factors were not uniform. Fifth, no prospective cohort study was included, which precluded a causal relationship. Last but not the least, only one study has specified the ratio of omega-3 and omega-6 PUFAs consumption (17), some issues could not be addressed. These limitations might weaken the significance of this study.

## Conclusions

Our results suggested that the dietary omega-3 PUFAs level was inversely associated with sarcopenia. However, current evidence is still insufficient to demonstrate the definite relationship between dietary omega-6 PUFAs levels and sarcopenia. More well-designed prospective cohort studies with the dietary omega-3/omega-6 PUFAs ratio are still needed.

## Data Availability Statement

Publicly available datasets were analyzed in this study. This data can be found here: PubMed, Web of Science, Embase.

## Author Contributions

YZ conceived the idea, performed the statistical analysis, and drafted this meta-analysis. HG and JL selected and retrieved relevant papers. WX and YL assessed each study. YZ and YL were the guarantors of the overall content. All authors revised and approved the final manuscript.

## Funding

This work was supported by the National Key R&D Program of China (2019YFA0111900), National Natural Science Foundation of China (Nos. 81874030, 82072506, and 82102581), National Postdoctoral Science Foundation of China (2021M693562), National Clinical Research Center for Geriatric Disorders (Xiangya Hospital, 2021KFJJ02), National Clinical Research Center for Orthopedics, Sports Medicine and Rehabilitation (2021-NCRC-CXJJ-PY-40), Hunan Young Talents of Science and Technology (No. 2021RC3025), Provincial Outstanding Postdoctoral Innovative Talents Program of Hunan (2021RC2020), Provincial Natural Science Foundation of Hunan (No. 2020JJ3060 and 2019JJ40517), Provincial Clinical Medical Technology Innovation Project of Hunan (No. 2020SK53709), Innovation-Driven Project of Central South University (No. 2020CX045), Wu Jieping Medical Foundation (320.6750.2020-03-14), Young Investigator Grant of Xiangya Hospital, Central South University (2020Q14), and FuQing Postdoc Program of Xiangya Hospital, Central South University (176).

## Conflict of Interest

The authors declare that the research was conducted in the absence of any commercial or financial relationships that could be construed as a potential conflict of interest.

## Publisher's Note

All claims expressed in this article are solely those of the authors and do not necessarily represent those of their affiliated organizations, or those of the publisher, the editors and the reviewers. Any product that may be evaluated in this article, or claim that may be made by its manufacturer, is not guaranteed or endorsed by the publisher.

## References

[1] Cruz-Jentoft A Baeyens J Bauer J Boirie Y Cederholm T Landi F . Sarcopenia: European consensus on definition and diagnosis: report of the European Working Group on Sarcopenia in Older People. Age Ageing. (2010) 39:412–23. 10.1093/ageing/afq034 20392703 PMC2886201

[2] Cruz-Jentoft A Sayer A. Sarcopenia. Lancet. (2019) 393:2636–46. 10.1016/S0140-6736(19)31138-9 31171417

[3] Marcos-Pardo P González-Gálvez N López-Vivancos A Espeso-García A Martínez-Aranda L Gea-García G . Sarcopenia, diet, physical activity and obesity in European middle-aged and older adults: the LifeAge study. Nutrients. (2020) 13:8. 10.3390/nu13010008 33375058 PMC7822002

[4] Bowen T Schuler G Adams V. Skeletal muscle wasting in cachexia and sarcopenia: molecular pathophysiology and impact of exercise training. J Cachexia Sarcopenia Muscle. (2015) 6, 197–207. 10.1002/jcsm.12043 26401465 PMC4575550

[5] Vatic M Haehling S Ebner N. Inflammatory biomarkers of frailty. Exp Gerontol. (2020) 133:110858. 10.1016/j.exger.2020.110858 32007546

[6] Bloom I Shand C Cooper C Robinson S Baird J. Diet Quality and sarcopenia in older adults: a systematic review. Nutrients. (2018) 10:308. 10.3390/nu10030308 29510572 PMC5872726

[7] Yao W Li J Wang J Zhou W Wang Q Zhu R . Effects of dietary ratio of n-6 to n-3 polyunsaturated fatty acidss on immunoglobulins, cytokines, fatty acids composition, and performance of lactating sows and suckling piglets. J Anim Sci Biotechnol. (2012) 3:43 10.1186/2049-1891-3-43 23270637 PMC3598561

[8] Djoussé L Akinkuolie A Wu J Ding E Gaziano J. Fish consumption, omega-3 fatty acidss and risk of heart failure: a meta-analysis. Clin Nutr. (2012) 31:846–53. 10.1016/j.clnu.2012.05.010 22682084 PMC3509256

[9] Jang H Park K. Omega-3 and omega-6 polyunsaturated fatty acidss and metabolic syndrome: a systematic review and meta-analysis. Clin Nutr. (2020) 39:765–73. 10.1016/j.clnu.2019.03.032 31010701

[10] Sadeghi O Djafarian K Ghorabi S Khodadost M Nasiri M Shab-Bidar S . Dietary intake of fish, n-3 polyunsaturated fatty acidss and risk of hip fracture: a systematic review and meta-analysis on observational studies. Crit Rev Food Sci Nutr. (2019) 59:1320–33. 10.1080/10408398.2017.1405908 29244536

[11] Candow D Forbes S Little J Cornish S Pinkoski C Chilibeck P. Effect of nutritional interventions and resistance exercise on aging muscle mass and strength. Biogerontology. (2012) 13:345–58. 10.1007/s10522-012-9385-4 22684187

[12] Huang Y Chiu W Hsu Y Lo Y Wang Y. Effects of omega-3 fatty acidss on muscle mass, muscle strength and muscle performance among the elderly: a meta-analysis. Nutrients. (2020) 12:3739. 10.3390/nu12123739 33291698 PMC7761957

[13] Son Y Lee S Kim S An W. Omega-3 fatty acids modulates molecules associated with sarcopenia in muscle of 5/6 nephrectomy rats. J Am Soc Nephrol. (2018) 29:956.

[14] Lee S Jeong E Son S Choi H Son Y Kim S . Effect of omega-3 fatty acids and menaquinone-7 on osteopenia and sarcopenia inadenineand low protein diet induced uremic rats. Nephrol Dial Transpl. (2018) 33:i162–3. 10.1093/ndt/gfy104.FP384

[15] Choi H Lee S Son S Kim K Son Y Kim S . Combined treatment with cholecalciferol and omega-3 fatty acids modulates molecules associated with sarcopenia and cardiac hypertrophy in 5/6 nephrectomy rats. J Am Soc Nephrol. (2017) 28:209.27335120

[16] Borg S de Groot L Mijnarends D Vries J Verlaan S Meijboom S . Differences in nutrient intake and biochemical nutrient status between sarcopenic and nonsarcopenic older adults-results from the maastricht sarcopenia study. J Am Med Dir Assoc. (2016) 17:393–401. 10.1016/j.jamda.2015.12.015 26825685

[17] Okamura T Hashimoto Y Miki A Kaji A Sakai R Iwai K . Reduced dietary omega-3 fatty acids intake is associated with sarcopenia in elderly patients with type 2 diabetes: a cross-sectional study of KAMOGAWA-DM cohort study. J Clin Biochem Nutr. (2020) 66:233–7. 10.3164/jcbn.19-85 32523250 PMC7263935

[18] Das A Cumming R Naganathan V Blyth F Couteur D Handelsman D . Associations between nutrient intakes and dietary patterns with different sarcopenia definitions in older Australian men: the concord health and ageing in men project. Public Health Nutr. (2020) 24:378–83. 10.1017/S1368980020004528 33243306 PMC10195399

[19] Yang W Lee J Kim Y Lee J Kang H. Increased omega-3 fatty acids intake is inversely associated with sarcopenic obesity in women but not in men, based on the 2014–2018 Korean National Health and Nutrition Examination Survey. J Clin Med. (2020) 9:3856. 10.3390/jcm9123856 33260970 PMC7761316

[20] Reis A Limirio L Santos H de Oliveira E. Intake of polyunsaturated fatty acidss and ω-3 are protective factors for sarcopenia in kidney transplant patients. Nutrition. (2021) 81:110929. 10.1016/j.nut.2020.110929 32745708

[21] Otsuka Y Iidaka T Horii C Muraki S Oka H Nakamura K . Dietary intake of vitamin E and fats associated with sarcopenia in community-dwelling older japanese people: a cross-sectional study from the fifth survey of the ROAD study. Nutrients. (2021) 13:1730. 10.3390/nu13051730 34065253 PMC8161000

[22] Liberati A Altman DG Tetzlaff J Mulrow C Gøtzsche PC Ioannidis JP . The PRISMA statement for reporting systematic reviews and meta-analyses of studies that evaluate healthcare interventions: explanation and elaboration. BMJ. (2009) 339:b2700. 10.1136/bmj.b2700 19622552 PMC2714672

[23] Begg C Mazumdar M. Operating characteristics of a rank correlation test for publication bias. Biometrics. (1994) 50:1088–101. 10.2307/2533446 7786990

[24] Gingras A White P Chouinard P Julien P Davis T Dombrowski D . Long-chain omega-3 fatty acidss regulate bovine whole-body protein metabolism by promoting muscle insulin signalling to the Akt-mTOR-S6K1 pathway and insulin sensitivity. J Physiol. (2007) 579:269–84. 10.1113/jphysiol.2006.121079 17158167 PMC2075371

[25] Smith G Atherton P Reeds D Mohammed B Rankin D Rennie M. Dietary omega-3 fatty acids supplementation increases the rate of muscle protein synthesis in older adults: a randomized controlled trial. Am J Clin Nutr. (2011) 93:402–12. 10.3945/ajcn.110.005611 21159787 PMC3021432

[26] Simopoulos A. An increase in the omega-6/omega-3 fatty acids ratio increases the risk for obesity. Nutrients. (2016) 8:128. 10.3390/nu8030128 26950145 PMC4808858

[27] Baillie R Takada R Nakamura M Clarke S. Coordinate induction of peroxisomal acyl-CoA oxidase and UCP-3 by dietary fish oil: A mechanism for decreased body fat deposition. Prostaglandins Leukot Essent. Fat Acids. (1999) 60:351–6. 10.1016/S0952-3278(99)80011-8 10471120

[28] Ukropec J Reseland J Gasperikova D Demcakova E Madsen L Berge R. The hypotriglyceridemic effect of dietary n-3 FA is associated with increased β-oxidation and reduced leptin expression. Lipids. (2003) 38:1023–9. 10.1007/s11745-006-1156-z 14669966

[29] Sakai C Ishida M Ohba H Yamashita H Uchida H Yoshizumi M . Fish oil omega-3 polyunsaturated fatty acidss attenuate oxidative stress-induced DNA damage in vascular endothelial cells. PLoS ONE. (2017) 12:e0187934. 10.1371/journal.pone.0187934 29121093 PMC5679535

[30] Okamura T Hashimoto Y Osaka T Fukuda T Hamaguchi M Fukui M. The sodium-glucose cotransporter 2 inhibitor luseogliflozin can suppress muscle atrophy in Db/Db mice by suppressing the expression of foxo1. J Clin Biochem Nutr. (2019) 65:23–8. 10.3164/jcbn.18-114 31379410 PMC6667382

[31] Santiago E Roriz A Ramos L Ferreira A Oliveira C Gomes-Neto M. Comparison of calorie and nutrient intake among elderly with and without sarcopenia: a systematic review and meta-analysis. Nutr Rev. (2021) 79:1338–52. 10.1093/nutrit/nuaa145 33616172

[32] BorgS LuikingY HelvoortA BoirieY ScholsJ de GrootC. Low levels of branched chain amino acids, eicosapentaenoic acid and micronutrients are associated with low muscle mass, strength and function in community-dwelling older adults. J Nutr Health Aging. (2019) 23:27–34. 10.1007/s12603-018-1108-330569065

[33] Jang I Jung H Park J Kim J Lee S Lee E . Lower serum n-3 fatty acids level in older adults with sarcopenia. Nutrients. (2020) 12:2959. 10.3390/nu12102959 32992568 PMC7600475

[34] Murphy R Mourtzakis M Chu Q Reiman T Mazurak V. Skeletal muscle depletion is associated with reduced plasma (n-3) fatty acidss in non-small cell lung cancer patients. J Nutr. (2010) 140:1602–6. 10.3945/jn.110.123521 20631325

[35] Belury M Cole R Andridge R Keiter A Raman S Lustberg M . Erythrocyte long-chain ω-3 fatty acidss are positively associated with lean mass and grip strength in women with recent diagnoses of breast cancer. J Nutr. (2021) 151:2125–33. 10.1093/jn/nxab109 34036350 PMC8349126

[36] Stark K Van Elswyk M Higgins M Weatherford C Salem N. Global survey of the omega-3 fatty acidss, docosahexaenoic acid and eicosapentaenoic acid in the blood stream of healthy adults. Prog Lipid Res. (2016) 63:132–52. 10.1016/j.plipres.2016.05.001 27216485

[37] Ferreri C Masi A Sansone A Giacometti G Larocca A Menounou G . Fatty acids in membranes as homeostatic, metabolic and nutritional biomarkers: recent advancements in analytics and diagnostics. Diagnostics. (2016) 7:1. 10.3390/diagnostics7010001 28025506 PMC5373010

[38] Cheng P Huang W Bai S Wu Y Yu J Zhu X . BMI affects the relationship between long chain N-3 polyunsaturated fatty acids intake and stroke risk: a meta-analysis. Sci Rep. (2015) 5:14161. 10.1038/srep14161 26369699 PMC4572932

[39] HanP ZhaoJ GuoQ WangJ ZhangW ShenS . Incidence, risk factors, and the protective effect of high body mass index against sarcopenia in suburb-dwelling elderly Chinese populations. J Nutr Health Aging. (2016) 20:1056–60. 10.1007/s12603-016-0704-327925147

[40] Harden C Dible V Russell J Garaiova I Plummer S Barker M . Long-chain polyunsaturated fatty acids supplementation had no effect on body weight but reduced energy intake in overweight and obese women. Nutr Res. (2014) 34:17–24. 10.1016/j.nutres.2013.10.004 24418242

[41] Cho Y Lim Y Yun J Yoon H Park M. Sex- and age-specific effects of energy intake and physical activity on sarcopenia. Sci Rep. (2020) 10:9822. 10.1038/s41598-020-66249-6 32555196 PMC7300112

[42] DiNicolantonio J O'Keefe J. Importance of maintaining a low omega-6/omega-3 ratio for reducing inflammation. Open Heart. (2018) 5:e000946. 10.1136/openhrt-2018-000946 30564378 PMC6269634

[43] Ramsden C Zamora D Leelarthaepin B Majchrzak-Hong S Faurot K Suchindran C . Use of dietary linoleic acid for secondary prevention of coronary heart disease and death: evaluation of recovered data from the Sydney Diet Heart Study and updated meta-analysis. BMJ. (2013) 346:e8707. 10.1136/bmj.e8707 23386268 PMC4688426

[44] DiNicolantonio J O'Keefe J. Importance of maintaining a low omega-6/omega-3 ratio for reducing platelet aggregation, coagulation and thrombosis. Open Heart. (2019) 6:e001011. 10.1136/openhrt-2019-001011 31218005 PMC6546183

